# Growth, Biochemical Characteristics, Flesh Quality, and Gut Microbiota of the Pacific White Shrimp (*Penaeus vannamei*) Fed a Defatted Superworm (*Zophobas atratus*) Larvae Meal

**DOI:** 10.1155/2023/8627246

**Published:** 2023-07-08

**Authors:** Hongxing Lin, Xiaolong Liang, Fenglu Han, Xiaolong Luo, Erchao Li

**Affiliations:** ^1^Key Laboratory of Tropical Hydrobiology and Biotechnology of Hainan Province, Hainan Aquaculture Breeding Engineering Research Center, College of Marine Sciences, Hainan University, Haikou, Hainan 570228, China; ^2^School of Life Sciences, East China Normal University, Shanghai 200241, China

## Abstract

This study evaluated the effects of defatted superworm (*Zophobas atratus*) larvae meal (DBWLM) as an alternative protein ingredient for juvenile Pacific white shrimp (*Penaeus vannamei*). Six isonitrogenous and isolipidic experimental diets were characterized by replacing 0%, 15%, 30%, 45%, 60%, and 75% fish meal (DBWLM0, DBWLM15, DBWLM30, DBWLM45, DBWLM60, and DBWLM75, respectively) with DBWLM on a w/w basis and feeding them to juvenile shrimp (0.34 ± 0.04 g) for 56 days. The results showed that the replacement of up to 75% fish meal by DBWLM had no negative effect on the growth performance of *P*. *vannamei*. The survival of shrimp in the DBWLM30 group was the highest, and the weight gain, specific growth rate, feed conversion ratio, condition factor, and apparent digestibility coefficients of dry matter in the DBWLM15 group were the highest. The substitution of DBWLM for fish meal significantly increased the elasticity of flesh, improved the total content of umami amino acids in flesh (aspartic acid, glutamic acid, glycine, and alanine), promoted lipid metabolism in shrimp, and reduced serum lipid levels. With the increase in DBWLM level, serum acid phosphatase, alkaline phosphatase activity, and intestinal inflammatory gene expression (IGF-1 and IL-6) were inhibited, malondialdehyde content decreased, and total antioxidant capacity level and superoxide dismutase activity increased significantly. Histological sections of the hepatopancreas showed that when 60% or more fish meal was replaced, the hepatopancreas atrophied and had irregular lumen distortion, but the cell membrane was not damaged. Microbiome analysis showed that the abundance of Bacteroidetes and Firmicutes increased and the abundance of Proteobacteria decreased in the DBWLM replacement group, and it was rich in “metabolism”-related functional pathways. It is worth mentioning that the expression of amino-acid-related enzymes was upregulated in the DBWLM15 and DBWLM30 groups, and the DBWLM75 group inhibited the biosynthesis of steroids and hormones. To conclude, the replacement of 15%–45% fish meal with DBWLM can result in better growth and immune status, improved meat elasticity, and reduced inflammation in *P*. *vannamei*. However, it is recommended that the replacement level should not exceed 60%, otherwise it will cause atrophy of hepatopancreas cells.

## 1. Introduction

Pacific white shrimp (*Penaeus vannamei*) is popular for its thin shell, rich nutritional value, high meat content, and deliciousness [1]. It is one of the major economic shrimp species in the global aquaculture industry [2] and is also the main consumer of fish meal [3]. It is well known that fish meal is a good source of aquatic feed protein and plays a key role in maintaining aquatic animal yield, growth, and general health functions [4–6]. However, the expansion of shrimp production, the deterioration of the marine environment, and the reduction in fishery resources have led to a decrease in fish meal production and an increase in prices. Therefore, the search and development of alternative protein sources based on renewable natural resources is an urgent need for the sustainable development of the aquafeed industry [7–10].

At present, various studies have shown that the plant proteins have potential problems, such as antinutritional factors [11, 12], low palatability [13], gastrointestinal inflammation [14], and the pressure of cultivated land use [15]. Compared with plant protein, insect protein is closer to fish meal in terms of nutritional composition and is considered to be a high-quality protein source to replace fish meal. Insect proteins have great nutritional and sustainable natural characteristics, and their large resources have been considered potential new sustainable protein sources [16, 17]. The European Commission has approved the use of processed insect proteins in aquafeeds (Regulation 2017/893/EC, 2017). According to a survey, insect protein sources generally contain high-crude lipid content [7, 18], which is prone to lipid peroxidation and limits the amount to be added to feed, and some studies have demonstrated that their fatty acid profiles are species-specific and too high in monounsaturated fatty acids to be suitable for feed use [16, 19]. It is worth noting that the fatty acid composition in feed affects the fat composition in animals, which in turn affects the quality and health characteristics of aquatic products, such as muscle [20, 21]. Muscle chewiness of European sea bass (*Dicentrarchus labrax*) improved with increasing levels of defatted yellow mealworms replacing a fish meal, indicating that a defatted insect diet may improve the quality of fish fillets [22]. Researchers have previously reported that defatting by physical screw pressing can produce different combinations of free amino acids, increasing protein content and reducing excess harmful fats [23, 24], thus reducing the risk of fat oxidation that may cause liver damage in animals [25]. To date, defatted insect species to replace fish meal have shown prospects in the aquafeed industry; for example, the replacement of 64% fish meal by defatted black soldier fly larvae did not alter the growth performance and intestinal morphology of Japanese seabass (*Lateolabrax japonicus*) [26]. Defatted silkworm pupa powder completely replacing fish meal does not affect the growth of *P*. *vannamei*, but it is recommended that the substitution level be limited to 75% because complete substitution leads to atrophy of hepatopancreatic cells [27]. Replacement of 80% of dietary fish meal with defatted *Tenebrio molitor* larvae meal did not affect European bass's food intake or central balance [28].

Most studies related to insect substitution for fish meal have focused on black soldier flies and yellow mealworms. However, the superworm (*Zophobas atratus*), also known as barley worm, is a species that is easy to reproduce, easy to raise, vigorous, and productive [29]. Superworm powder contains a high proportion of amino acids, unsaturated fatty acids, vitamins, and mineral elements, and a small amount of biologically active substances can also be extracted from it [8, 30, 31]. To date, superworms have been studied mainly in biodegradation [32–34]. At present, the effect of DBWLM replacing fish meal on *P*. *vannamei* has not been systematically studied. Therefore, this study evaluated the effects of DBWLM replacing fish meal on the growth, biochemical, flesh quality, and gut microbiota of *P*. *vannamei* to explore the potential of DBWLM as a fish meal substitute in shrimp diets.

## 2. Materials and Methods

### 2.1. Diet Preparation

Six isonitrogenous and isolipidic diets were formulated in the experiment, and DBWLM was added to the diets at 0% (DBWLM0), 15% (DBWLM15), 30% (DBWLM30), 45% (DBWLM45), 60% (DBWLM60), and 75% (DBWLM75) to replace fish meal in the basal diets, corresponding to dietary inclusion levels of 0%, 4.15%, 8.30%, 12.45%, 16.60%, and 20.75%. The ingredient composition and approximation analysis of all experimental diets are shown in Table 1. The defatted superworm larvae meal was purchased from a commercial supplier (Hunan Haikun Agricultural Science and Technology Co., Ltd). The defatted superworm larvae meal is composed of 94.80% dry matter, 60.58% crude protein, 13.10% crude fat, and 5.0% ash. All dry ingredients were crushed and weighed according to the proportion of feed formulation, gradually diffused, and fully mixed. Pellets with a diameter of 1.5 mm were extruded in a twin-screw screw extruder (F-26, SCUT, Guangdong Industrial Plant, China). The diets were dried indoors until the moisture content was below 10%, and the feeds were crushed into pellets suitable for shrimp at different growth stages, sealed in plastic bags, and stored at −20°C until use. The amino acid composition of all experimental diets is shown in Table 2.

### 2.2. Experimental Shrimp and Feeding Trial

*P*. *vannamei* post larvae were obtained from a local shrimp hatchery in Dongfang, Hainan, China. All juvenile *P*. *vannamei* were acclimated in the experimental environment for 4 weeks. Juvenile shrimp (initial weight: 0.34 ± 0.04 g) were selected and randomly divided into six groups (with four replicates per group) in cylindrical tanks (200 L 60 cm diameter × 55 cm high) with 25 shrimp per tank. During the feeding trial (8 weeks), the parameters of rearing seawater quality were water temperature (28–30°C), ammonia nitrogen (0–0.2 mg/L), pH 7.9–8.2, and dissolved oxygen (5–6 mg/L). About 60% (90 L) of the culture water (150 L) was replaced at 10 : 30 daily. The shrimp were fed four times a day (07:00, 12:00, 17:00, and 22:00). The daily feeding amount is to achieve apparent satiation within half an hour. One hour after feeding, feces were collected by the siphon method. Selected intact enveloped feces were freeze-dried, ground up, and sealed in self-sealing bags and stored in the refrigerator to measure apparent digestibility. The system of dirt was cleaned promptly to maintain water quality. Water temperature, feeding amount, and mortality were recorded.

### 2.3. Sample Collection and Analysis

#### 2.3.1. Sample Collection

At the end of 8 weeks of culture experiment, the shrimp were fasted for 24 hr for sample collection. The shrimp were placed on ice for anesthesia for 10 min, and the total weight of each barrel was weighed and counted to calculate the specific growth rate (SGR), survival, and feed coefficient. Then, the barrels were individually weighed and body length was measured. Three shrimp were randomly selected from each barrel at −20°C and used to determine whole body composition. Shrimp heart blood was drawn into 1.5 mL centrifuge tubes using a sterile disposable syringe, placed in a refrigerator at 4°C for 24 hr, and centrifuged at 3,000 r/min for 10 min at 4°C, and the supernatant was stored at −80°C for the determination of serum biochemical parameters. The hepatopancreas and intestinal tissues of three shrimp were randomly collected and fixed in 4% paraformaldehyde to prepare HE-stained sections. Two to three segments of abdominal muscle with square shapes were taken on ice plates and placed in 5 mL cryotubes for the determination of muscle texture characteristics. The hepatopancreas and intestinal tissue were placed in 2 mL cryotubes, immediately placed in liquid nitrogen, and then placed in a −80°C refrigerator until use. All analyses were performed in six replicates. This experimental protocol was approved by the Animal Experiment Ethics Committee of Hainan University (Issue No. HNUAUCC-2021-00119).

#### 2.3.2. Determination of Indicators

Growth parameters were calculated using the following equations:

- Survival (%) = 100 × (final number of shrimp/initial number of shrimp)
- Weight gain (WG, %) = 100 × (final weight − initial weight)/initial weight
- SGR (%day^−1^) = 100 × (Ln (final weight) − Ln (initial weight))/days
- Feed conversion ratio (FCR) = dry weight of diet consumed/total WG
- Condition factor (CF, g/cm^3^) = 100 × shrimp weight/shrimp length^3^
- Hepatosomatic index (HSI, %) = 100 × (weight of hepatopancreas/weight of shrimp)

The apparent digestibility coefficients (ADCs) of dry matter and protein for the diets were calculated using the following formula [35, 36].

- ADC of dry matter (%) = 100 × (1− (dietary Cr_2_O_3_ level/feces Cr_2_O_3_ level) × (feces dry matter level/dietary dry matter level))
- ADC of crude protein (%) = 100 × (1− (dietary Cr_2_O_3_ level/fecal Cr_2_O_3_ level) × (fecal crude protein level/dietary crude protein level))

#### 2.3.3. Analysis Method of Nutritional Components

The analysis of crude protein, crude lipid, moisture and ash in whole shrimp, diets, and feces was performed according to the standard method of the Association of Official Analytical Chemists [37]. Crude protein and crude lipid were determined by the Kjeldahl method (KjeltecTM8200, FOSS, Sweden) and Soxhlet extraction method (FOSS, SoxtecTM2055, Switzerland), respectively. The moisture content was dried at 105°C to constant weight (24 hr). Ash content was determined by the muffle method at 550°C (PCD-E3000, Serials, Peaks, Japan). The free amino acid composition of the diets, shrimp meat, and defatted superworm larvae meals was determined by an automatic amino acid analyzer (Hitachi L-8900) according to the national standard method (GB/T 18246-2019). The content of Cr_2_O_3_ in the diets and feces was determined by an inductively coupled plasma emission spectrometer (ICP-OES, Prodigy7 LEEMAE LABS, USA).

#### 2.3.4. Muscle Texture Characteristics

About 12 shrimp from each treatment group were studied, and the third abdominal segment of the shrimp body was uniformly removed and cut into square samples for testing. Muscle texture characteristics were measured by an MS-PRO high-precision texture analyzer. After adjusting the intensity and height of the instrument, the samples were tested with a flat-bottomed cylindrical probe p/5 (5 mm diameter) test mode: TPA (Texture Profile Analysis) mode. The test conditions were as follows: pretest rate of 3 mm/s, test rate of 1 mm/s, posttest rate of 1 mm/s, compression degree of 50%, dwell time of 5 s, load-bearing probe type of Auto5 g, and data collection rate of 200. Each sample was tested three times and averaged. The textural parameters tested included hardness, viscosity, cohesiveness, elasticity, gumminess, chewiness, and resilience.

#### 2.3.5. Serum Immunological Assays

According to the method of Lopez [38], the activities of acid phosphatase (ACP) and alkaline phosphatase (AKP) were measured. The total antioxidant capacity (T-AOC) activity was detected according to the method of Javahery et al. [39]. Nitric oxide (NO) activity was measured by an NO assay kit (Microwell plate method). The above indexes were determined by commercial kits (Nanjing Jiancheng Institute of Biological Engineering, Nanjing, China), and the specific experimental operations were performed according to the kit instructions.

#### 2.3.6. Biochemical Indexes and Digestive Enzymes

Hepatopancreatic tissues were homogenized as described above and centrifuged at 3,500 × *g* for 15 min at 4°C, and the supernatant was collected in a new sterile tube. Triglyceride (TG), total cholesterol (T-CHO), T-AOC, catalase (CAT), malondialdehyde (MDA), glutathione peroxidase (GSH-Px), and superoxide dismutase (SOD) were determined using a commercial kit from Nanjing Jiancheng Bioengineering Institute. The intestinal and hepatopancreatic tissues were added to 0.9% ice–cold sterile saline, and 10 volumes (v/w) of homogenates were prepared and centrifuged at 1,000 × *g* (5415R, Eppendorf, Germany) for 15 min at 4°C. The supernatants were collected, and the activities of total protein (TP), amylase, lipase, and trypsin were determined. The above indexes were measured according to the kit instructions produced by Nanjing Jiancheng Institute of Biological Engineering.

#### 2.3.7. Hepatopancreas Histology

Three shrimp were randomly selected from each replicate, and a total of 12 shrimp were selected from each group. The hepatopancreas tissue was immediately placed in centrifuge tubes containing 4% paraformaldehyde. After 24 hr of fixation, they were dehydrated, washed with chloroform, and embedded in solid wax blocks. The solid wax blocks were cut into 5 *μ*m slices using a rotary microscope, stained with hematoxylin and eosin (H&E), observed, and photographed under an optical microscope (Eclipse 200, Nikon, Japan).

#### 2.3.8. Gut Inflammatory Factors

After homogenization of intestinal tissues, the supernatant was taken for the determination of tumor necrosis factor-*α* (TNF-*α*), interleukin-6 (IL-6), and insulin-like growth factor-I (IGF-I) by shrimp enzyme-linked immunosorbent assay (ELISA) kits. The above kits were purchased from Shanghai Jianglai Biotechnology Co., Ltd., and the determination was carried out in strict accordance with the instructions of the kits.

#### 2.3.9. Gut Microbiota Analysis

Four intestinal tissues were randomly selected from each group and mixed into one sample, with five samples (DBWLM0, DBWLM15, DBWLM30, and DBWLM75) per group for gut microbial analysis. The amount of extracted DNA was measured using a NanoDrop ND-2000 spectrophotometer (Thermo Fisher Scientific, Waltham, MA, USA), and the quality of DNA was detected by agarose gel electrophoresis. The V3–V4 region of the bacterial 16 S rRNA gene was amplified by PCR (GeneAmp 9700, ABI, USA) using the forward primer 338 F (5'-ACTCCTACGGGAGGCAGCA-3') and the reverse primer 806R (5'-GGACTACHVGGGTWTCTAAT-3'). The purified amplicons were sequenced isochronously and in pairs (2 × 300) on the Illumina MiSeq platform (Illumina, San Diego, USA) according to the standard protocol of Shanghai Personal Biotechnology Co. Sequence data were analyzed using QIIME2 and the R package (v3.2.0). The alpha diversity index at the amplicon sequence variant (ASV) level Chao1 [40], observational species, Shannon [41], and Simpson [42] were calculated using the ASV table in QIIME2. Beta diversity analysis was performed using the Bray‒Curtis metric to study the structural changes in microbial communities among samples [43]. Visualization was performed using principal coordinate analysis (PCoA), nonmetric multidimensional scale (NMDS), and unweighted pair group method [44] with arithmetic mean (UPGMA) hierarchical clustering. The phylogenetic investigation (PICRUSt) of communities was performed using unobserved state reconstruction to standardize the OTU (operational taxonomic units) table to eliminate the influence of 16 S marker genes on the genome copy number of species. Kyoto Encyclopedia of Genes and Genomes Ortholog (KO) information was obtained from each OTU corresponding to the gene ID. The abundance of metabolic pathway information was obtained at three levels based on the Kyoto Encyclopedia of Genes and Genomes (KEGG) database. The sequences obtained in the present study were deposited in the Sequence Read Archive of National Center for Biotechnology Information database under the collection number PRJNA895828.

### 2.4. Statistical Analysis

Normality test was performed before one-way analysis of variance was performed on the variables of the six groups in this experiment to assess whether there was a significant difference between the observed responses (*P* < 0.05). Tukey's honestly significant difference (Tukey's HSD) test was used to test whether there were significant differences between the groups. Otherwise, orthogonal polynomials were used to determine whether the response was linear and/or quadratic for subsequent trend analysis. All data were analyzed using SPSS Statistics 23 IBM, Armonk, NY, USA). GraphPad Prism eight software was used to plot histograms of experimental data. Experimental results are expressed as the mean ± standard error (mean ± SE).

Spearman correlation analysis of gut microbiota with biochemical indicators (such as ACP, AKP, NO, T-AOC, SOD, MDA, GSH-Px, and CAT) was performed using the online data analysis platform Gene Cloud (https://www.genescloud.cn).  ^*∗*^Mark microbial–biochemical indicators with significant indigenous associations (*P* < 0.05),  ^*∗*^indicates *P* < 0.05,  ^*∗∗*^indicates *P* < 0.01.

## 3. Results

### 3.1. Growth Performance, Body Composition, Apparent Digestibility Coefficients, and Digestive Enzyme Activities

The growth indexes of shrimp at different DBWLM levels are shown in Table 3. After DBWLM substitution of fish meal, linear and quadratic responses of FCR and CF were observed (*P* < 0.05). Compared with the control group, the FCR and CF of shrimp were significantly increased in all substitution groups (*P* < 0.05), the FBW, WG, and SGR of the DBWLM 15 group were significantly increased (*P* < 0.05), and the survival was significantly higher in the DBWLM30 group (*P* < 0.05). After replacing fish meal with DBWLM, the HSI of shrimp in each group showed a significant downward trend (*P* < 0.05). DBWLM significantly promoted the ADC of dry matter in the shrimp feed when the content of DBWLM exceeded 15% (*P* < 0.05). The ADC of crude protein of DBWLM diets did not differ significantly from the control diet (Table 3). As shown in Table 4, there was a linear and quadratic effect of dietary DBWLM level on crude lipid content (*P* < 0.05). The crude lipid content of shrimp showed a significant downward trend (*P* < 0.05) after DBWLM replaced fish meal. There were no significant differences in the crude protein, moisture, and ash among all the treatments (*P* > 0.05).

Hepatopancreas lipase activity was significantly higher in the DBWLM45 and DBWLM60 groups than in the control group (*P* < 0.05, Table 5). There was no significant difference in trypsin and amylase activities in the hepatopancreas of shrimp among all the treatments (*P* > 0.05, Table 5). The intestinal trypsin activity of shrimp first increased and then decreased as the fish meal replacement level increased. In addition, intestinal trypsin activity was significantly decreased in the DBWLM60 and DBWLM75 groups compared to the control group (*P* < 0.05, Table 5). However, the intestinal lipase and amylase activities were not significantly different between the groups (*P* > 0.05, Table 5).

### 3.2. Flesh Texture Analysis, Amino Acid Composition, and Intestinal Inflammatory Factors

In terms of flesh texture (Table 6), after the DBWLM substitution of fish meal, shrimp muscle elasticity increased significantly and reached the highest level in the DBWLM15 group (*P* < 0.05). The hardness of the DBWLM15 group was significantly higher than that of the control group and tended to decrease with increasing substitution levels (*P* < 0.05). There was no significant difference in adhesiveness, gumminess, cohesiveness, chewiness, or resilience among the groups (*P* > 0.05). A total of 16 free amino acids were detected in the shrimp meat (Table 7), and no significant differences were observed between the test groups in terms of nonessential amino acids and total amino acids in shrimp muscle (*P* > 0.05). The content of arginine was the highest among the essential amino acids. The levels of glutamic acid and lysine showed a significant quadratic relationship with dietary DBWLM levels (*P* < 0.05). Serine and glutamic acid were significantly reduced in the DBWLM30 group compared to the control group (*P* < 0.05). In addition, the total contents of taste amino acids (aspartate, glutamate, glycine, and alanine) in the DBWLM 15 group were better than those in the other groups.

After replacing fish meal with DBWLM, the contents of IGF-1 and IL-6 showed significant linear and quadratic response trends (Table 8, *P* < 0.05). With the increase of DBWLM substitution level, the contents of IL-6 and IGF-1 increased first and then decreased (*P* < 0.05). There was no significant difference in TNF-*α* levels between the treatment groups (*P* > 0.05).

### 3.3. Serum and Hepatopancreas Biochemical Parameters

After DBWLM replaced fish meal, serum TG, T-CHO, and hepatopancreas T-CHO levels showed a downward trend, reaching the lowest level in the DBWLM15 group (Table 9). Hepatopancreas TG in DBWLM75 group was significantly lower than that in DBWLM0 group (*P* < 0.05, Table 9). Both linear and secondary responses were evident for hepatopancreas TG after DBWLM replaced fish meal (*P* < 0.05).

As shown in Table 10, the dietary DBWLM level had significant linear and quadratic effects on the serum NO contents and ACP and AKP activities of shrimp (*P* < 0.05). Compared with the control group, the serum NO contents of DBWLM45, DBWLM60, and DBWLM75 were significantly increased (*P* < 0.05), and conversely, their serum AKP activity decreased significantly (*P* < 0.05). With increasing DBWM replacement level, serum ACP activity showed a significant downward trend (*P* < 0.05).

In terms of antioxidant capacity (Table 10), the dietary DBWLM level had significant linear and quadratic effects on the hepatopancreas T-AOC, SOD, and GSH-PX activities of shrimp (*P* < 0.05). With the increase of DBWM replacement level, the activities of T-AOC and SOD showed a significant upward trend (*P* < 0.05), whereas the activities of CAT and GSH-PX decreased significantly (*P* < 0.05). There was no significant difference in MDA among all of the treatments (*P* > 0.05).

### 3.4. Hepatic Histomorphology

**Figure 1:**
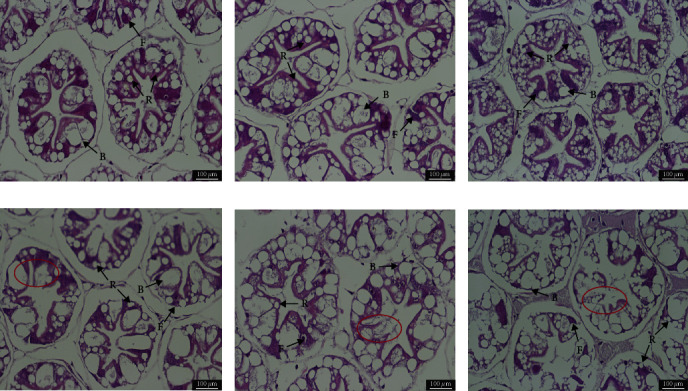
Hepatopancreas of shrimp stained with H&E and fed six different diets. (a) DBWLM0, ×400; (b) DBWLM15, ×400; (c) DBWLM30, ×400; (d) DBWLM45, ×400; (e) DBWLM60, ×400; (f) DBWLM75, ×400. B, blasenzellen cells (*B* cells) usually contain a large secretory small vesicle; F, fibrous cells (*F* cells) contain a large number of ribosomes and a well-developed endoplasmic reticulum; R, restzellen cells (*R* cells) usually contain lipid vacuoles of variable size.

From the tissue sections (Figure 1), it can be seen that dietary DBWLM substitution of fish meal below 45% did not cause damage to the hepatopancreas tissue structure of shrimp, with closely arranged hepatopancreas cells and intact cell membranes, whereas the hepatopancreas of the DBWLM60 and DBWLM75 groups atrophied and had irregular lumen deformation, fat vesicles grew larger, but the cell membranes remained intact and unbroken.

### 3.5. Gut Microbiota Analysis

An average of 105,272 sequences were detected in each intestinal microbial sample, and 93,972 valid sequences were obtained by denoising or clustering. The coverage rate of each sample was above 99%, indicating that the sequencing depth fully met the requirements of bacterial community analysis.

#### 3.5.1. Gut Microbiota Composition and Diversity

**Figure 2:**
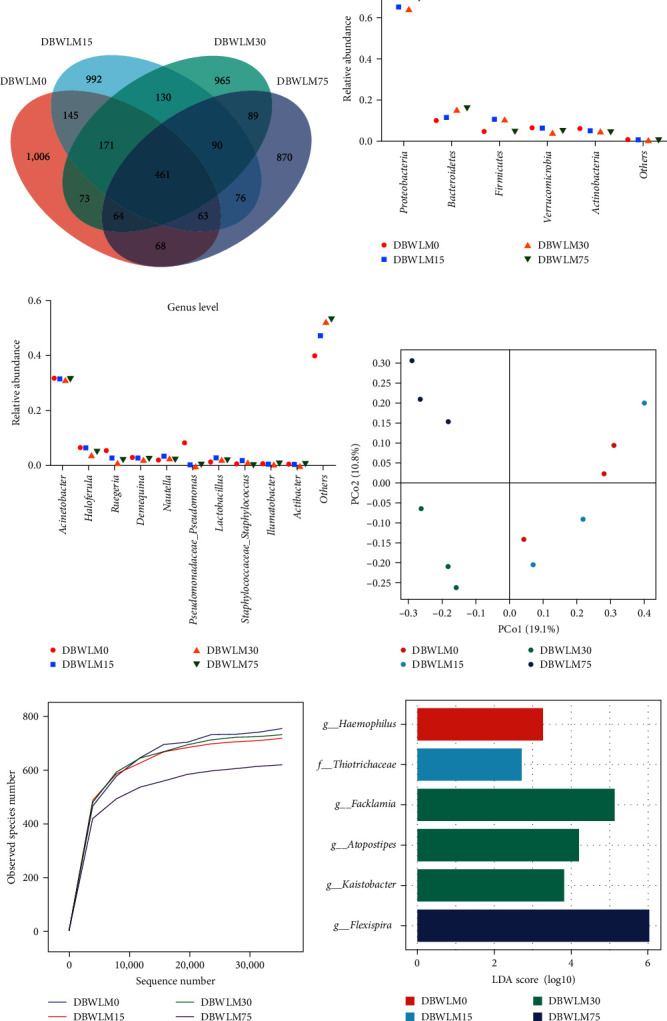
Gut microbial community composition of *P*. *vannamei* fed DBWLM instead of fish meal. (a) Venn diagram showing the number of unique and shared OTUs. (b) The average relative abundance of the top five dominant phyla levels. (c) The average relative abundance of the top 10 dominant genera. (d) Principal coordinate analysis (PCoA). Each point represents a sample and points of the same color belong to the same group. (e) A sparse curve diagram measures the diversity of each sample to a certain extent. (f) Histogram of the difference-rich classification group (LEfSe, LDA > 3.5).

A total of 3401 OTUs were obtained, of which the control and DBWLM groups had 461 identical OTUs, accounting for 13.5% of the total OTUs (Figure 2(a)). Among them, the control group had the highest number of OTUs, and the number of OTUs gradually decreased with increasing substitution level. As shown in Figure 2(b), the most important phylum in all samples was Proteobacteria, followed by Bacteroidetes, Firmicutes, Verrucomicrobia, and Actinobacteria. The abundance of Bacteroidetes progressively increased with increasing levels of DBWLM in the diet. At the genus level, *Acinetobacter* and *Haloferula* were the dominant bacteria (Figure 2(c)). PCoA was used to assess the overall difference in bacterial community structure based on Bray‒Curtis distances. The results showed that different levels of DBWLM replacing fish meal in the diet resulted in different gut microbial community compositions (Figure 2(d)). The smoother the curve, the more uniform the colony distribution, indicating that the sequencing results were sufficient to reflect the diversity of the current samples (Figure 2(e)). LEfSe analysis showed that *Atopostipes*, *Facklamia*, and *Kaistobacter* were significantly enriched in the DBWLM30 group (Figure 2(f)).

**Figure 3:**
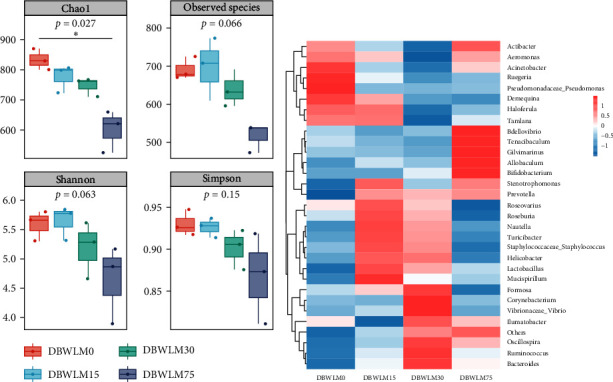
Changes in alpha diversity and heatmap of gut microbial community composition of *P*. *vannamei* fed DBWLM as an alternative to fish meal. (a) Alpha diversity indexes (Chao1, ACE, Shannon, Simpson). (b) The relative abundance is a thermogram of the top 30 genera, and a warmer color indicates a higher abundance of species.

The *α*-diversity analysis showed that the DBWLM0 and DBWLM15 groups had the highest Shannon and Simpson indexes, and the Chao1 index was significantly lower than that of the control group (Figure 3(a)). As shown in Figure 3(b), a heatmap revealed that dietary DBWLM altered the relative abundance of intestinal genera (the top 30) in each group. In the control group, two genera, *Ruegeria* and *Pseudomonas*, had the highest relative abundance. In the DBWLM15 group, only one genus, *Mucispirillum*, had the highest relative abundance. In the DBWLM30 group, *Corynebacterium* and *Vibrio* had the highest relative abundance. In the DBWLM75 group, five genera, *Bdellovibrio*, *Tenacibaculum*, *Gilvimarinus*, *Allobaculum*, and *Bifidobacterium*, had the highest relative abundance.

#### 3.5.2. Gut Microbiota Functional Prediction

**Figure 4:**
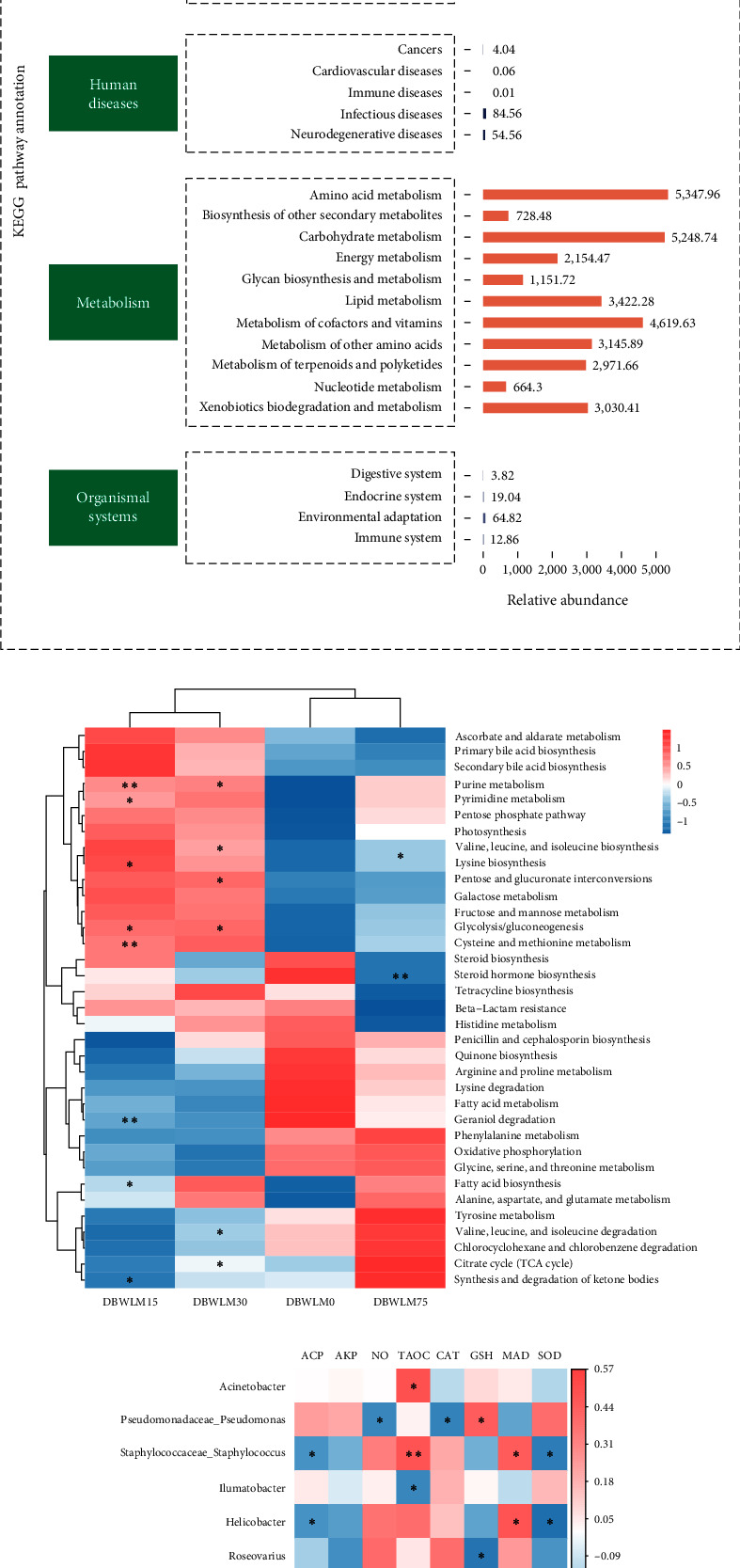
Effect of feeding different levels of DBWLM instead of fish meal on gut microflora function of *P*. *vannamei* and correlation analysis of biochemical indexes. (a) Level-2 function prediction of intestinal microbial abundance (KEGG metabolic pathway statistics). (b) Clustering heatmap with relatively rich KEGG level-3 functions. (c) Correlation analysis between intestinal flora and biochemical indexes. The illustration shows the correlation coefficient value. The default red represents a positive correlation and the blue represents a negative correlation. The star number between different patches indicates the importance of each exposure compared with the control. Statistical analysis using a *t* test,  ^*∗*^represents *P* < 0.05,  ^*∗∗*^represents *P* < 0.01.

All groups of enriched KEGG pathway statistics at the KEGG-1 level were divided into six categories: “cellular processes,” “environmental information processing,” “genetic information processing,” “human diseases,” “metabolism,” and “organismal systems.” Metabolic function is the most important KEGG pathway. At the KEGG2 level, metabolic functions mainly included “amino acid metabolism,” “carbohydrate metabolism,” “fat metabolism,” and “cofactor and vitamin metabolism” (Figure 4(a)). At the KEGG3 level, “amino acid-related enzyme metabolism” was significantly increased in the DBWLM15 and DBWLM30 groups compared with the control group (*P* < 0.05, Figure 4(b)).

#### 3.5.3. Association Analysis of Gut Microbiota and Biochemical Indexes

Association analysis between the intestinal microbiota at the genus level and biochemical indicators is shown in Figure 4(c). The abundances of *Acinetobacter*, *Staphylococcus*, and *Roseburia* were positively correlated with the content of T-AOC (*P* < 0.05). In contrast, the contents of *Ilumatobacter* and *Bdellovibrio* were negatively correlated with the activity of T-AOC (*P* < 0.05). The abundances of *Staphylococcus*, *Helicobacter*, and *Roseburia* were positively correlated with the activity of MDA (*P* < 0.05). The abundance of *Staphylococcus*, *Helicobacter*, and *Roseburia* was negatively correlated with the activity of ACP (*P* < 0.05). The abundance of *Pseudomonas*, *Aeromonas*, and *Mucispirillum* was negatively correlated with the activity of CAT (*P* < 0.05). The abundance of *Staphylococcus*, *Helicobacter*, and *Roseburia* was negatively correlated with the activity of SOD (*P* < 0.05). The abundance of *Pseudomonas* was negatively correlated with the activity of NO (*P* < 0.05). The activity of GSH-Px was positively correlated with the abundance of *Pseudomonas* and negatively correlated with the abundance of *Roseovarius* (*P* < 0.05).

#### 3.5.4. Gut Microbiota Network and Correlation Analysis

**Figure 5:**
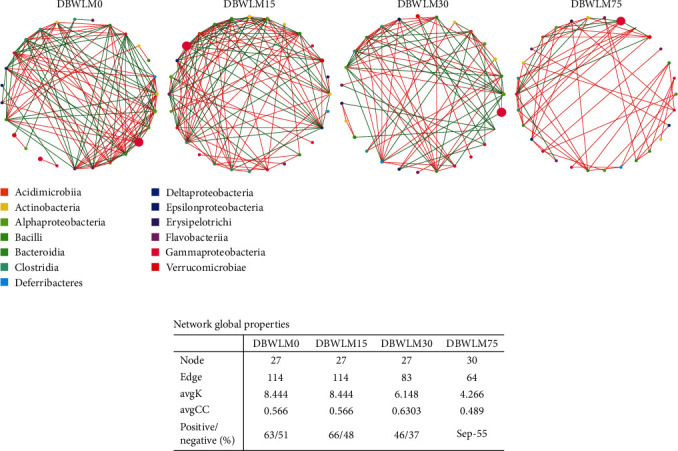
Interaction network analysis of the gut microbial community. (a) Interspecies interaction network of intestinal microbiota for *P*. *vannamei* fed with different DBWLM levels. (b) Network properties. The red line is positively correlated, the green line represents a negative correlation, and each node represents a genus. Node color indicates that they belong to different classes. Source: Rahimnejad et al. [27].

The effects of dietary DBWLM on the interspecific interaction network of the gut microbial community were analyzed (Figures 5(a) and 5(b)). Compared with the control group, the DBWLM75 group showed an increase in the number of nodes but a decrease in the number of edges. The control and DBWLM15 groups had the same number of nodes and edges, and the largest average degree (avgK) and average clustering coefficient (avgCC) were the largest, indicating that the control and DBWLM15 groups had more connected gut microbial community networks, the most complex interspecies relationships, and the largest positive interactions.

## 4. Discussion

### 4.1. Growth

Insect meal is considered to be the most-promising candidate for fish meal in aquaculture [29, 45–47]. The palatability, nutritional availability, and digestibility of insect meal have been improved to a certain extent by defatting [48]. A meta-analysis showed that defatted insects improved the growth performance of species fed appropriate levels of substitution [49]. Our study showed that the substitution of 75% fish meal with DBWLM did not negatively affect the growth performance of *P*. *vannamei* and that substitution of 15% fish meal with DBWLM promoted WG and FCR of *P*. *vannamei*. It is possible that defatting and the addition of methionine to supplement the amino acid balance of the diet are the main factors that improve the growth performance of shrimp. Various studies on the substitution of fish meal by defatted insect meal have been reported extensively. A significant improvement in the growth characteristics of *P*. *vannamei* shrimp was observed when 75% FM was replaced by defatted yellow mealworm (*Tenebrio molitor*) [50]. Similar results were obtained in a study by Wang et al. [26], who confirmed that defatted black soldier fly meal replacement diets fish meal up to 64% level did not affect the growth performance of *P*. *vannamei*, and defatted silkworm (*Bombyx mori* L.) pupae meal replacement with 100% fish meal did not influence *P*. *vannamei* growth [27]. Therefore, the appropriate level of substitution may be different due to different insect species, processing technology, feed formulation, feeding period, and rearing conditions [51, 52].

Chromium oxide is commonly used as an exogenous indicator in nutritional studies to determine apparent digestibility [53]. Rahimnejad et al. [27] measured that the apparent digestibility of dry matter in the diets of *P*. *vannamei* was improved after replacing fish meal with a defatted silkworm pupae meal. Similarly, the results of the apparent digestibility of dry matter in this study showed that *P*. *vannamei* could effectively utilize DBWLM. Digestive enzyme activity is well known to reflect potential effects on feed utilization and growth performance [54, 55]. In the present study, feeding DBWLM instead of fish meal increased the hepatopancreas lipase activity of *P*. *vannamei*, but no significant changes in trypsin and amylase were observed. This may be because the superworm meal was defatted to affect the *n*-3 long-chain polyunsaturated fatty acid content [56]. Similar results were found in a study of gilthead seabream (*Sparus aurata*) [57]. In contrast, Rios et al. [58] found that *Tenebrio molitor*, which replaces fish meal in the diet, did not affect the trypsin, chymotrypsin, and amylase activities of *P*. *vannamei*. Similarly, the same results were found for the effect of *Hermetia illucens* larvae meal on the digestive enzymes of Jian carp [59]. Therefore, it is speculated that the findings of different studies may be related to the widely varying nutrient composition of insect protein sources, fish species, and feeding habits.

### 4.2. Muscle Texture and Amino Acid Composition

It has been reported that the intake of different diets can change the quality of shrimp meat, such as texture features [60, 61]. In this study, the muscle texture and amino acid composition of shrimp were investigated. After replacing fish meal with DBWLM, the muscles showed good elasticity, which could be due to the formation of a tighter network structure of muscle proteins and their hydrated layers. However, a significant decrease in hardness was observed when the substitution level was 75%, which is consistent with the findings of Martínez-Córdova et al. [62]. It is speculated that fish with higher fat or water content will reduce the structural elements of muscle tissue, thereby reducing its hardness [63]. In addition, it is worth mentioning that the freshness of meat depends on the content of flavor amino acids, including aspartic acid, glutamic acid, glycine, and alanine [64, 65]. The results of this study showed that DBWLM increased the total amount of flavor amino acids in shrimp muscle after replacing fish meal, and the essential amino acids in shrimp muscle remained relatively balanced in all groups. Therefore, DBWLM could provide essential amino acids to shrimp for optimal growth, and DBWLM replacement of fish meal increased the content of flavor amino acids in muscle.

### 4.3. Immunity, Intestinal Inflammation, and Hepatopancreatic Health

Serum parameters can reflect the physiological stress response of fish to nutritional and environmental changes [66]. Dietary defatted insect meal could avoid undesirable effects by reducing the content of saturated fatty acids and improving the quality of protein [7, 67]. In this study, TG and T-CHO levels in the serum and hepatopancreas of shrimp were lower than those in the control group after replacing fish meal with DBWLM. DBWLM may facilitate hemolymph transport of cholesterol to the hepatopancreas, promote lipid metabolism, and reduce lipid levels in the body. This finding is consistent with the results of using dietary defatted black soldier fly meal instead of fish meal on Japanese seabass [26] and defatted black soldier fly meal in sword carp [59]. Similarly, in other fish, processing methods such as defatting and fermentation can lead to a decrease in cholesterol levels [27, 68, 69]. Furthermore, the results of crude fat in shrimp body composition in this study verified this view. Notably, the chitin contained in insect meals may induce intestinal inflammation [70]. To further study the effect of dietary DBWLM on the health of shrimp, nonspecific immune indexes and intestinal inflammatory factors were examined in shrimp serum. ACP and AKP are hydrolases in lysosomes that eliminate foreign substances [71, 72]. In the present experiment, the activities of ACP and AKP in serum were inhibited with increasing DBWLM levels, and when the substitution amount exceeded 45%, the contents of intestinal inflammatory cytokines (IL-6 and IGF-1) showed a downward trend, which indicated that DBWLM improved the intestinal inflammatory response and reduce the stress response of shrimp. A previous study found that dietary defatted black soldier fly meal replacement of fish meal did not produce inflammatory events in the zebrafish hepatopancreas [73]. The results of this experiment showed that the structure of the hepatopancreas was not damaged at 15%–45% substitution level. Meantime, Choi et al. [74] reported that complete replacement of fish meal by *Tenebrio molitor* had no effect on the hepatopancreas and intestinal tissue of *P*. *vannamei*.

### 4.4. Antioxidant

In general, the antioxidant properties of chitin and its derivatives in insect meals have been repeatedly reported [75–78]. It is well known that T-AOC is an important indicator of the antioxidant system to remove excessive reactive oxygen species (ROS) [79, 80], and MDA is commonly used as a marker of oxidative damage [81]. In addition, key antioxidant enzymes, such as CAT, SOD, and GSH-Px, can reduce oxidative stress damage in organisms [67, 82]. Although there was no significant difference in MDA content among the groups, the MDA content in the high-level DBWLM supplementation group had an upward trend.

It shows that too high substitution level may lead to oxidative stress in shrimp. This result may be related to the fact that DBWLM contains chitin. It has been reported that an appropriate amount of chitin and other similar immunoactive substances can improve the body's antioxidant properties and enhance immune and other biological functions [83, 84]. However, excessive use of DBWLM with high levels of chitin content may cause damage to the body [85, 86] such as atrophy of the hepatopancreas of shrimp, and increased lipid peroxidation products such as MDA. With the increase in the DBWLM replacement fish meal level, the CAT and GSH-Px activities decreased, which indicated that there was still a risk of oxidative stress in the high-level DBWLM supplementation group. It has been reported that the vulnerability of H_2_O_2_ can be explained by the amount of CAT, which is associated with preventing lipid peroxidation [87]. However, the T-AOC levels increased significantly with the increase in the DBWLM replacement fish meal level. When the body responds to the risk of oxidative damage, there is also an adaptive increase in the activity of some antioxidant enzymes, thereby reducing lipid peroxidation, which confirms the antioxidant capacity of this insect meal [88]. Similarly, Rahimnejad et al. [27] reported that feeding an appropriate amount of defatted silkworm pupa meal instead of fish meal enhanced the T-AOC of *P*. *vannamei* and reduced MDA concentrations to promote antioxidant capacity. Furthermore, in sword carp, after the appropriate amount of defatted silkworm pupa powder replaced fish meal, the SOD activity in the liver gradually increased, and the MDA concentration gradually decreased [68]. These studies show that defatted insect powder also needs to be controlled in an appropriate amount in the feed to avoid causing oxidative damage to animals, and the appropriate level of addition can improve the antioxidant capacity of shrimp.

### 4.5. Gut Microbial Composition

The gut microbiota has been shown to be influenced by dietary composition [89, 90]. In the present study, significant changes in gut microbial community structure were observed in PCoA, similar to previous studies on shrimp [91, 92]. This result suggests that the replacement of fish meal by dietary DBWLM reshaped the gut microbiota structure of shrimp. The results of the Venn diagram showed that 461 bacterial members were shared between the different DBWLM substitution groups, suggesting that these bacterial members may be the core microbiota in the gut of shrimp. The abundance of gut microbiota is an important factor affecting energy absorption and storage [93]. At the genus level, the abundances of *Thiotrichaceae*, *Atopostipes*, and *Flexispira* were significantly increased in the DBWLM15, DBWLM30, and DBWLM75 groups, respectively. The role of *Thiotrichaceae* in the intestine is currently unknown. *Atopostipes* have a certain regulatory function on the reproductive function of animals [94]. *Flexispira* is closely related to *Helicobacter* pylori, which is pathogenic to the body [95]. This result may indicate that excessive DBWLM replacement levels may influence the health status of the shrimp intestinal flora.

Pseudomonas can inhibit pathogenic bacteria and could be a potential source of probiotics for shrimp culture [96]. In this study, correlations between the intestinal flora and biochemical parameters were analyzed. Pseudomonas was negatively correlated with NO and CAT and positively correlated with GSH-Px, indicating that DBWLM can improve growth performance and the antibacterial immune system by regulating intestinal flora structure. KEGG functional prediction showed that dietary DBWLM substitution of fish meal enriched “metabolism”-related functional pathways, such as “amino acid metabolism,” “carbohydrate metabolism,” and “cofactor and vitamin metabolism.” Therefore, DBWLM can regulate the metabolic functions of the flora, and promote the stability of the intestinal microbial community.

The results of the present study showed that the amino-acid-related enzymes in the DBWLM15 and DBWLM30 groups enhanced interspecific interactions of the gut microbiota, but the DBWLM75 group inhibited the biosynthesis of steroids and hormones, and these changes may improve gut health in *P*. *vannamei*. It is worth noting that the gut microbiota affects host metabolism through an ecological network formed by interspecific interactions [97, 98]. The control group and the DBWLM15 group had the most connections and the most complex ecological network, indicating that the composition of the intestinal flora of the two groups of shrimp remained in a relatively stable state. However, the relationship between the microecological network of the gut and shrimp health still needs further study.

## 5. Conclusion

The replacement of up to 75% fish meal by DBWLM did not have any negative effect on the growth performance of *L.vannamei*. Notably, in the current study, the shrimp obtained better growth and immunity, improved meat elasticity, and reduced inflammation when the replacement ratio was less than 45%. This study mainly highlights the feasibility and appropriate level of replacement of DBWLM as a new protein source in shrimp diets. The substitution of 15%–45% fish meal with DBWLM is beneficial to improve the economic and ecological benefits of shrimp culture. However, DBWLM substitution of 60%–75% fish meal has devastating effects on hepatopancreatic tissue structure and intestinal health. Therefore, it is recommended that the substitution level should not exceed 60%.

## Figures and Tables

**Table 1:** Ingredient composition (dry basis, g/kg) and proximate analysis of experimental diets fed to *P*. *vannamei*.

Ingredients	Experiment diets					
	DBWLM0	DBWLM15	DBWLM30	DBWLM45	DBWLM60	DBWLM75
Fish meal	250	212.5	175	137.5	100	62.5
Defatted *Zophobas atratus* larvae meal	0	41.5	83	124.5	166	207.5
Soybean meal	210	210	210	210	210	210
Peanut meal	120	120	120	120	120	120
Shrimp shell powder	100	100	100	100	100	100
Wheat meal	130	130	130	130	130	130
Fish oil	10	11	11	12	12	12
Soybean oil	35	30	25	17	12	8
Soyabean lecithin	15	15	15	15	15	15
Cholesterol	5	5	5	5	5	5
Methionine	1.5	2.5	3	3.5	4	4.5
Calcium dihydrogen phosphate	10	10	10	10	10	10
Vitamin premix^1^	20	20	20	20	20	20
Mineral premix^2^	20	20	20	20	20	20
Vitamin C ester	2	2	2	2	2	2
Binder	30	30	30	30	30	30
Choline chloride	5	5	5	5	5	5
Antioxidant BHT	0.5	0.5	0.5	0.5	0.5	0.5
Chromium trioxide	0.5	0.5	0.5	0.5	0.5	0.5
Betaine	10	10	10	10	10	10
Microcrystalline cellulose	25.5	24.5	25	27.5	28	27.5
Total	1000	1000	1000	1000	1000	1000
Proximate analyses (% dry weight)						
Crude protein (%)	40.576	40.710	40.799	41.061	40.507	40.868
Crude lipid (%)	8.543	8.653	8.586	8.659	8.677	8.702
Ash (%)	12.77	13.307	13.282	13.193	13.594	13.832
Moisture (%)	11.08	11.34	10.89	11.19	11.04	10.98

^1^One kilogram of vitamin premix contained: vitamin A: 4800 IU; vitamin D_3_ 8000IU; l-ascorbic acid-2-polyphosphate 35% activity C: 35.71 g; folic acid: 0.18 g; biotin: 0.05 g; riboflavin: 3.0 g; DL-Ca-pantothenic acid: 5.0 g; pyridoxine HCl B_6_: 1.0 g; thiamin HCl: 0.5 g; vitamin B_12_: 0.002 g; Menadione K_3_: 2.0 g; DL-*α*-tocopherol acetic acid: 20IU; nicotinic acid: 5.0 g; inositol: 5 g. ^2^One kilogram of mineral premix contained: zinc sulfate monohydrate: 20.585 g; calcium iodate: 0.117 g; copper sulfate pentahydrate: 0.625 g; manganese sulfate monohydrate: 1.625 g; magnesium sulfate monohydrate: 39.860 g; cobalt chloride: 0.010 g; ferrous sulfate monohydrate: 11.179 g; calcium hydrogen phosphate dihydrate: 166.442 g.

**Table 2:** Amino acid composition of the six experimental diets (dry matter basis, g/kg).

Ingredients	Experiment diets						
	DBWLM0	DBWLM15	DBWLM30	DBWLM45	DBWLM60	DBWLM75	DBWLM
Essential amino acids (EAAS)							
Arginine	23.9	24.7	24.4	23.2	22.7	24.8	35.1
Histidine	8.3	8.7	8.7	8.6	8.4	9.4	20.7
Isoleucine	14.1	14.7	14.5	13.9	13.6	15.0	28.1
Leucine	24.5	25.5	25.0	24.0	23.3	25.4	45.6
Lysine	16.7	17.0	16.7	15.7	15.1	16.5	37.1
Methionine	7.6	7.8	7.5	7.1	6.9	7.0	53.0
Phenylalanine	21.5	23.0	23.9	24.3	24.0	27.2	49.9
Threonine	14.1	14.5	14.3	13.6	13.3	14.4	26.4
Valine	16.2	17.1	17.2	16.9	16.5	18.4	39.0
Nonessential amino acids (NEAAS)							
Aspartate	35.9	37.1	36.2	34.7	33.9	36.7	43.4
Alanine	18.6	19.6	19.7	19.1	18.7	20.8	55.6
Glutamate	56.0	58.6	57.8	55.4	54.3	59.3	89.7
Glycine	20.2	20.8	20.4	19.1	18.7	20.0	30.2
Proline	15.6	16.5	16.8	16.7	16.4	18.3	35.6
Tyrosine	12.8	14.3	15.3	16.4	16.1	19.3	51.7
Serine	15.7	16.4	16.3	15.8	15.4	17.0	27.8
Total essential amino acids (TEAAS)	146.9	153.0	152.2	147.3	143.8	158.1	287.2
Total nonessential amino acids (TNEAAS)	174.8	183.3	182.0	177.2	173.5	198.6	334.0
Total amino acids (TAAS)	321.7	336.3	334.2	324.5	317.3	356.7	621.2

**Table 3:** Growth performance and digestibility of *P*. *vannamei* fed six different experimental diets for 8 weeks.

Items	Experiment diets						Pr > *F*		
	DBWLM0	DBWLM15	DBWLM30	DBWLM45	DBWLM60	DBWLM75	ANOVA	Linear	Quadratic
IBW (g)	0.48 ± 0.02	0.49 ± 0.03	0.47 ± 0.02	0.47 ± 0.05	0.45 ± 0.03	045 ± 0.03	0.008	0.193	0.531
FBW(g)	8.57 ± 0.21^b^	9.20 ± 0.35^a^	8.37 ± 0.28^b^	8.87 ± 0.22^b^	8.16 ± 0.18^c^	8.04 ± 0.26^c^	0.002	0.010	0.066
WG (%)	4110.76 ± 459.32^b^	4754.40 ± 354.37^a^	4121.73 ± 436.80^b^	4120.15 ± 320.62^b^	4090.90 ± 369.35^b^	4071.98 ± 328.56^b^	0.010	0.350	0.644
FCR	1.65 ± 0.17^c^	1.82 ± 0.10^a^	1.84 ± 0.12^ab^	1.79 ± 0.08^ab^	1.75 ± 0.13^b^	1.72 ± 0.12^b^	< 0.001	0.001	0.002
SGR (%day^−1^)	6.44 ± 0.22^b^	6.68 ± 0.21^a^	6.47 ± 0.16^b^	6.47 ± 0.10^b^	6.47 ± 0.15^b^	6.46 ± 0.12^b^	0.007	0.296	0.370
HSI (%)	5.80 ± 0.16^a^	4.65 ± 0.19^c^	5.34 ± 0.30^ab^	5.23 ± 0.33^b^	5.12 ± 0.39^b^	5.03 ± 0.36^bc^	0.001	0.173	0.236
Survival (%)	80.42 ± 4.20^b^	81.26 ± 3.62^b^	86.64 ± 3.95^a^	84.47 ± 3.32^ab^	82.50 ± 4.05^ab^	80.58 ± 4.21^b^	0.007	0.704	0.067
CF (g/cm^3^)	83.82 ± 3.29^b^	89.98 ± 2.72^a^	90.19 ± 2.03^a^	92.99 ± 1.73^a^	92.28 ± 1.47^a^	90.56 ± 2.34^a^	< 0.001	0.002	< 0.001
ADCDM (%)	73.41 ± 1.33^b^	79.68 ± 2.04^a^	76.82 ± 1.72^a^	75.28 ± 1.67^ab^	75.82 ± 1.64^ab^	75.29 ± 1.59^ab^	< 0.001	0.001	0.008
ADCCP (%)	84.12 ± 1.63	87.29 ± 2.89	86.43 ± 1.90	85.89 ± 1.83	85.12 ± 0.84	86.23 ± 1.30	0.034	0.134	0.439

*Note*. Growth parameters (initial body weight (IBW), final body weight (FBW), weight gain (WG), feed conversion ratio (FCR), specific growth rate (SGR), hepatosomatic index (HSI), survival, condition factor (CF), apparent digestibility coefficients of dry matter (ADCDM), and apparent digestibility coefficients of crude protein (ADCCP) were expressed as the mean ± standard error (*n* = 3). Different letters (a, b, c) in the same row represent significant differences (*P* < 0.05). Pr > *F*, significant probability associated with the *F*-statistic; ANOVA, analysis of variance.

**Table 4:** Body proximate composition of *P*. *vannamei* fed six different experimental diets for 8 weeks.

Composition (mg/kg)	Experiment diets						Pr > *F*		
	DBWLM0	DBWLM15	DBWLM30	DBWLM45	DBWLM60	DBWLM75	ANOVA	Linear	Quadratic
Crude protein	16.09 ± 0.62	15.91 ± 0.56	15.89 ± 0.61	16.98 ± 0.52	16.88 ± 0.65	16.93 ± 0.79	0.312	0.245	0.166
Crude lipid	4.50 ± 0.43^a^	4.08 ± 0.64^b^	3.77 ± 0.58^abc^	3.50 ± 0.47^abc^	2.91 ± 0.23^bc^	2.50 ± 0.13^c^	0.017	< 0.001	0.002
Moisture	75.76 ± 1.15	76.18 ± 1.34	75.94 ± 0.82	75.45 ± 0.78	75.45 ± 0.56	76.29 ± 1.28	0.438	0.993	0.890
Ash	2.17 ± 0.06	3.19 ± 0.02	3.98 ± 0.06	3.78 ± 0.08	2.58 ± 0.05	3.17 ± 0.01	0.318	0.204	0.344

*Note*. Data are presented as the mean ± standard error (*n* = 3). ANOVA, analysis of variance.

**Table 5:** Activity of trypsin, lipase, and amylase in the hepatopancreas and intestine of *P*. *vannamei* fed six different diets.

Items	Experiment diets						Pr > *F*		
	DBWLM0	DBWLM15	DBWLM30	DBWLM45	DBWLM60	DBWLM75	ANOVA	Linear	Quadratic
Intestinal									
Trypsase (U/mgprot)	93.39 ± 9.34^ab^	132.89 ± 9.89^a^	116.94 ± 9.66^ab^	86.86 ± 9.17^b^	64.17 ± 8.29^c^	58.65 ± 8.46^c^	0.002	< 0.001	0.001
Lipase (U/gprot)	88.09 ± 7.21	108.43 ± 6.78	88.08 ± 7.07	89.23 ± 7.91	89.54 ± 8.23	100.74 ± 9.85	0.408	0.017	0.619
Amylase (U/mgprot)	201.63 ± 23.12	213.51 ± 25.44	222.82 ± 23.65	189.37 ± 22.89	170.81 ± 22.61	136.16 ± 22.78	0.546	0.075	0.097
Hepatopancreas									
Trypsase (U/mgprot)	187.16 ± 16.28	189.88 ± 15.31	177.21 ± 17.24	173.59 ± 16.41	189.34 ± 17.32	189.14 ± 16.35	0.133	0.051	0.327
Lipase (U/gprot)	44.20 ± 5.47^c^	46.11 ± 6.40^bc^	61.45 ± 5.38^bc^	95.66 ± 6.45^a^	64.16 ± 5.56^b^	60.46 ± 7.50^bc^	0.003	0.010	0.038
Amylase (U/mgprot)	128.32 ± 15.29	133.26 ± 13.09	124.26 ± 9.25	126.77 ± 9.50	124.36 ± 13.43	125.37 ± 12.56	0.019	0.710	0.223

*Note*. Data are presented as the mean ± standard error (*n* = 3). ANOVA, analysis of variance.

**Table 6:** Muscle texture characteristics of *P*. *vannamei* fed six different experimental diets for 8 weeks.

Items	Experiment diets						Pr > *F*		
	DBWLM0	DBWLM15	DBWLM30	DBWLM45	DBWLM60	DBWLM75	ANOVA	Linear	Quadratic
Springiness	0.63 ± 0.12^b^	0.86 ± 0.10^a^	0.92 ± 0.06^a^	0.86 ± 0.11^a^	0.85 ± 0.12^a^	0.86 ± 0.10^a^	< 0.001	< 0.001	< 0.001
Adhesiveness (gf)	85.67 ± 7.39	80.72 ± 9.62	82.69 ± 8.95	84.86 ± 7.76	86.86 ± 8.75	89.18 ± 7.21	0.536	0.199	0.170
Gumminess (gf)	1410.12 ± 218.32	1277.68 ± 232.21	1185.27 ± 255.16	1102.57 ± 238.56	1225.85 ± 249.64	1241.85 ± 196.28	0.179	0.074	0.025
Hardness (gf)	569.77 ± 37.16^b^	582.59 ± 36.39^a^	564.62 ± 29.45^b^	540.16 ± 29.63^bc^	535.38 ± 30.69^bc^	528.18 ± 22.26^c^	0.032	0.006	0.024
Cohesiveness (gf)	0.45 ± 0.07	0.47 ± 0.06	0.46 ± 0.04	0.46 ± 0.07	0.46 ± 0.05	0.47 ± 0.06	0.912	0.550	0.782
Chewiness (gf)	950.95 ± 69.24	1089.61 ± 58.87	1105.07 ± 73.28	1142.79 ± 85.65	1055.81 ± 75.13	1001.28 ± 74.18	0.282	0.700	0.052
Resilience	0.31 ± 0.04	0.31 ± 0.07	0.31 ± 0.08	0.32 ± 0.08	0.34 ± 0.06	0.36 ± 0.03	0.352	0.025	0.059

*Note*. Data are presented as the mean± standard error (*n* = 3) Serum and hepatopancreas. ANOVA, analysis of variance.

**Table 7:** Muscle amino acid composition of *P*. *vannamei* fed six different experimental diets for 8 weeks (dry matter basis, g/kg).

Amino acids	Experiment diets						Pr > *F*		
	DBWLM0	DBWLM15	DBWLM30	DBWLM45	DBWLM60	DBWLM75	ANOVA	Linear	Quadratic
Essential amino acids (EAAS)									
Arginine	71.3 ± 1.7	71.5 ± 2.7	68.8 ± 2.5	71.3 ± 2.6	71.9 ± 2.6	74.9 ± 2.7	0.810	0.321	0.353
Histidine	20.4 ± 0.1	19.4 ± 0.1	18.6 ± 0.2	15.8 ± 0.1	19.2 ± 0.1	17.9 ± 0.2	0.305	0.060	0.172
Methionine	18.8 ± 0.1	21.2 ± 0.1	20.5 ± 0.2	21.4 ± 0.2	21.4 ± 0.1	19.6 ± 0.1	0.904	0.860	0.537
Isoleucine	35.0 ± 0.3	33.6 ± 0.3	32.0 ± 0.2	32.4 ± 0.2	33.4 ± 0.2	33.6 ± 0.1	0.582	0.672	0.181
Leucine	64.0 ± 0.4	61.2 ± 0.5	58.0 ± 0.5	59.0 ± 0.6	60.7 ± 0.5	61.6 ± 0.6	0.496	0.669	0.123
Lysine	60.8 ± 0.5	63.7 ± 0.5	60.0 ± 0.4	61.6 ± 0.6	64.0 ± 0.3	67.2 ± 0.5	0.319	0.816	0.046
Phenylalanine	36.7 ± 0.3	35.5 ± 0.3	33.6 ± 0.3	35.4 ± 0.2	34.7 ± 0.3	35.2 ± 0.1	0.710	0.555	0.456
Threonine	32.6 ± 0.2	30.8 ± 0.2	29.4 ± 0.1	30.0 ± 0.2	30.7 ± 0.1	31.5 ± 0.1	0.374	0.852	0.069
Valine	3.71 ± 0.03	3.56 ± 0.02	3.38 ± 0.02	3.46 ± 0.01	3.54 ± 0.01	3.57 ± 0.03	0.615	0.685	0.215
Nonessential amino acids (NEAAS)									
Aspartate	85.7 ± 2.3	82.3 ± 2.2	78.1 ± 2.4	80.1 ± 1.2	81.0 ± 2.2	82.3 ± 1.3	0.555	0.551	0.165
Alanine	59.8 ± 0.7	58.3 ± 0.9	57.4 ± 0.9	56.8 ± 1.0	56.6 ± 0.8	56.7 ± 0.8	0.978	0.395	0.646
Glutamate	152.0 ± 2.1^a^	140.6 ± 2.6^ab^	135.7 ± 3.0^b^	138.1 ± 3.4^ab^	140.2 ± 2.9^ab^	140.9 ± 3.8^ab^	0.034	0.336	0.090
Glycine	72.8 ± 0.4	69.6 ± 0.5	66.0 ± 0.5	69.6 ± 0.6	67.1 ± 0.4	71.4 ± 0.5	0.812	0.854	0.451
Proline	48.6 ± 0.4	46.9 ± 0.3	44.3 ± 0.5	44.1 ± 0.5	48.3 ± 0.3	49.6 ± 0.6	0.368	0.492	0.087
Tyrosine	31.7 ± 0.2	30.4 ± 0.4	29.0 ± 0.2	29.9 ± 0.3	30.0 ± 0.3	30.3 ± 0.2	0.666	0.570	0.259
Serine	32.0 ± 0.2^a^	29.6 ± 0.3^ab^	28.3 ± 0.2^b^	28.7 ± 0.2^b^	29.5 ± 0.2^ab^	30.4 ± 0.3^ab^	0.029	0.647	0.028
Total essential amino acids (TEAAS)	371.3 ± 4.3	372.5 ± 4.7	354.7 ± 4.0	361.5 ± 3.9	371.4 ± 4.1	377.2 ± 4.0	0.093	0.212	0.061
Total nonessential amino acids (TNEAAS)	482.6 ± 3.3	452.4 ± 2.8	438.8 ± 3.7	447.3 ± 4.2	452.7 ± 3.2	461.6 ± 3.4	0.082	0.392	0.082
Total amino acids (TAAS)	859.3 ± 6.0	830.2 ± 6.4	793.5 ± 6.5	809.8 ± 6.1	824.1 ± 5.8	838.8 ± 6.7	0.066	0.310	0.087

*Note*. Data are presented as the mean ± standard error (*n* = 3) Note: Data are presented as the mean ± standard error (*n* = 3). IGF-1, insulin-like growth factor-I; IL-6, interleukin-6; TNF-*α*, tumor necrosis factor-*α*; ANOVA, analysis of variance.

**Table 8:** Effects of different dietary DBWLM levels on the intestinal inflammatory reaction of *P*. *vannamei*.

Items	Experiment diets						Pr > *F*		
	DBWLM0	DBWLM15	DBWLM30	DBWLM45	DBWLM60	DBWLM75	ANOVA	Linear	Quadratic
IL-6 (pg/mL)	563.13 ± 53.62^ab^	631.90 ± 49.38^a^	689.01 ± 59.23^a^	676.86 ± 54.68^b^	642.25 ± 47.21^c^	581.15 ± 50.13^c^	0.019	0.003	0.005
IGF-1 (pg/mL)	746.72 ± 54.87^a^	779.01 ± 63.09^a^	794.47 ± 65.94^a^	601.07 ± 60.31^ab^	559.86 ± 48.72^ab^	430.68 ± 35.95^c^	0.001	0.001	0.032
TNF-*α* (pg/mL)	880.28 ± 72.13	966.97 ± 69.63	873.89 ± 70.14	755.26 ± 68.39	705.59 ± 69.19	692.17 ± 71.58	0.248	0.329	0.519

ANOVA, analysis of variance.

**Table 9:** TG and T-CHO content of *P*. *vannamei* fed six different experimental diets for 8 weeks.

Items	Experiment diets						Pr > *F*		
	DBWLM0	DBWLM15	DBWLM30	DBWLM45	DBWLM60	DBWLM75	ANOVA	Linear	Quadratic
Serum									
TG (mmol/L)	1.840 ± 0.210	1.430 ± 0.180	1.520 ± 0.230	1.610 ± 0.200	1.520 ± 0.180	1.520 ± 0.190	0.140	0.201	0.091
T-CHO (mmol/L)	2.378 ± 0.250	1.990 ± 0.190	2.217 ± 0.200	2.158 ± 0.180	2.130 ± 0.220	2.123 ± 0.210	0.230	0.181	0.340
Hepatopancreas									
TG (mmol/gprot)	0.418 ± 0.049^a^	0.353 ± 0.040^ab^	0.337 ± 0.051^ab^	0.335 ± 0.056^ab^	0.298 ± 0.041^ab^	0.270 ± 0.039^b^	0.029	0.011	0.002
T-CHO (mmol/gprot)	0.067 ± 0.007	0.043 ± 0.005	0.059 ± 0.007	0.058 ± 0.008	0.057 ± 0.007	0.048 ± 0.006	0.170	0.051	0.371

*Note*. Data are presented as the mean ± standard error (*n* = 3). TG, triglyceride; T-CHO, total cholesterol; ANOVA, analysis of variance.

**Table 10:** Serum biochemical indexes and hepatopancreas antioxidant capacity of *P*. *vannamei* fed six different experimental diets for 8 weeks.

Items	Experiment diets						Pr > *F*		
	DBWLM0	DBWLM15	DBWLM30	DBWLM45	DBWLM60	DBWLM75	ANOVA	Linear	Quadratic
Serum									
NO (*μ*mol/L)	24.45 ± 2.34^c^	25.66 ± 1.89^c^	26.90 ± 1.66^c^	28.99 ± 3.17^bc^	34.49 ± 2.98^ab^	37.96 ± 2.46^a^	< 0.001	< 0.001	< 0.001
ACP (U/dL)	28.29 ± 4.21^a^	23.10 ± 5.78^ab^	21.53 ± 5.64^b^	20.78 ± 5.91^bc^	14.68 ± 3.23^cd^	13.74 ± 3.05^d^	< 0.001	< 0.001	< 0.001
AKP (U/dL)	24.96 ± 3.12^a^	23.98 ± 3.44^ab^	23.70 ± 3.65^ab^	17.64 ± 2.89^abc^	15.64 ± 2.61^bc^	12.85 ± 2.78^c^	< 0.001	< 0.001	< 0.001
Hepatopancreas									
T-AOC (mmol/L)	2.97 ± 0.28^c^	3.21 ± 0.31^bc^	3.78 ± 0.38^bc^	4.47 ± 0.41^b^	4.64 ± 0.32^ab^	4.81 ± 0.35^a^	< 0.001	< 0.001	< 0.001
MDA (nmmol/mg prot)	3.33 ± 0.47	3.11 ± 0.40	3.28 ± 0.38	3.39 ± 0.45	3.46 ± 0.56	3.83 ± 0.50	0.908	0.315	0.482
CAT (U/mg prot)	21.96 ± 3.67^a^	18.41 ± 3.25^ab^	16.88 ± 3.06^ab^	16.32 ± 3.25^b^	15.47 ± 3.00^b^	14.50 ± 2.87^b^	0.079	0.002	0.006
SOD (U/mg prot)	216.25 ± 36.12^b^	237.90 ± 39.67^b^	297.89 ± 42.58^ab^	309.22 ± 35.89^ab^	323.20 ± 37.23^ab^	365.26 ± 42.60^a^	0.010	< 0.001	0.008
GSH-Px (U/mg prot)	417.46 ± 45.29^a^	345.79 ± 41.09^ab^	264.26 ± 39.25^ab^	261.13 ± 39.50^ab^	243.18 ± 40.43^ab^	196.37 ± 38.56^b^	0.013	< 0.001	0.003

*Note*. Data are presented as the mean ± standard error (*n* = 3). T-AOC, total antioxidant capacity; ACP, acid phosphatase; AKP, alkaline phosphatase; NO, nitric oxide; MDA, malondialdehyde; CAT, catalase; SOD, superoxide dismutase; GSH-Px, glutathione peroxidase; ANOVA, analysis of variance.

## Data Availability

All the data can be obtained in the manuscript and supplementary materials.
